# Friction massage versus kinesiotaping for short-term management of latent trigger points in the upper trapezius: a randomized controlled trial

**DOI:** 10.1186/s12998-017-0156-9

**Published:** 2017-09-12

**Authors:** Marzieh Mohamadi, Soraya Piroozi, Iman Rashidi, Saeed Hosseinifard

**Affiliations:** School of Rehabilitation Sciences, Shiraz University of Medical Sciences, Abiverdi 1 Street, Chamran Boulevard, Shiraz, Iran

**Keywords:** Latent, Trigger point, Trapezius, Pain threshold, Grip strength

## Abstract

**Background:**

Latent trigger points in the upper trapezius muscle may disrupt muscle movement patterns and cause problems such as cramping and decreased muscle strength. Because latent trigger points may spontaneously become active trigger points, they should be addressed and treated to prevent further problems. In this study we compared the short-term effect of kinesiotaping versus friction massage on latent trigger points in the upper trapezius muscle.

**Methods:**

Fifty-eight male students enrolled with a stratified sampling method participated in this single-blind randomized clinical trial (Registration ID: IRCT2016080126674N3) in 2016. Pressure pain threshold was recorded with a pressure algometer and grip strength was recorded with a Collin dynamometer. The participants were randomly assigned to two different treatment groups: kinesiotape or friction massage. Friction massage was performed daily for 3 sessions and kinesiotape was used for 72 h. One hour after the last session of friction massage or removal of the kinesiotape, pressure pain threshold and grip strength were evaluated again.

**Results:**

Pressure pain threshold decreased significantly after both friction massage (2.66 ± 0.89 to 2.25 ± 0.76; *P* = 0.02) and kinesiotaping (2.00 ± 0.74 to 1.71 ± 0.65; *P* = 0.01). Grip strength increased significantly after friction massage (40.78 ± 9.55 to 42.17 ± 10.68; *P* = 0.03); however there was no significant change in the kinesiotape group (39.72 ± 6.42 to 40.65 ± 7.3; *P* = 0.197). There were no significant differences in pressure pain threshold (2.10 ± 0.11 & 1.87 ± 0.11; *P* = 0.66) or grip strength (42.17 ± 10.68 & 40.65 ± 7.3; *P* = 0.53) between the two study groups.

**Conclusions:**

Friction massage and kinesiotaping had identical short-term effects on latent trigger points in the upper trapezius. Three sessions of either of these two interventions did not improve latent trigger points.

**Trial registration:**

Registration ID in IRCT: IRCT2016080126674N3.

## Background

Myofascial pain syndrome plays an important role in many types of chronic pain, and is considered one of the most common causes of muscle pain [1]. This syndrome is characterized by the presence of trigger points and tenderness in myofascial tissues. Trigger points are irritable spots in muscle fibers that produce pain on compression or stretching [2]. They are divided into two categories: active and latent. Latent trigger points do not cause local or referred pain until direct pressure is applied, but active trigger point cause pain even without manual pressure [3].

Active trigger points cause persistent pain that can result in movement restriction. This in turn may reduce muscle activity, strength and tonicity. Latent trigger points do not cause persistent pain; however they restrict movement, induce early fatigue and cause loss of tissue [4].

Latent trigger points may spontaneously become active trigger points. Inflammatory metabolites such as calcitonin gene-related peptide and substance P are found at the site of latent trigger points. These metabolites may reform latent trigger points and result in further pain and problems [5].

The upper trapezius muscle is the most common location of trigger points in the body, with six main trigger points identified thus far [6]. Any trigger point in the upper trapezius can cause neck stiffness, restricted cervical rotation and lateral flexion, shoulder elevation, neck pain and headache [3]. In addition, trigger points in the upper trapezius can affect grip strength, which depends on shoulder joint and scapula stability. The upper trapezius, lower trapezius and serratus anterior are the key muscles in scapular stability, and imbalances between these muscles can disrupt stability. Studies have shown that isometric exercising of these muscles in addition to shoulder stabilizers may increase grip strength. Thus, upper trapezius muscle dysfunction can reduce grip strength [7, 8].

Latent trigger points in the upper trapezius may disrupt muscle movement patterns and cause problems such as impingement syndrome, rotator cuff pathology and pain. They also cause cramping, decreased muscle strength, and changes in movement patterns and the timing of muscle activity [5]. In turn, the impact of trigger points in the upper trapezius on grip strength can affect physical performance, especially if these points spontaneously become active and cause additional problems. It is thus important to address latent trigger points and treat them to prevent further problems.

Among the various treatment methods proposed for trigger points, kinesiotaping is the least time consuming for patients. Because patients often do not believe it is important to treat latent trigger points, they may find kinesiotaping more acceptable given that it requires little time to implement. Despite the use of kinesiotaping in rehabilitation, the effect of this method for the treatment of trigger points remains unknown [2]. Moreover, it is worth noting that the physiological effects of kinesiotaping have never been tested in a high-quality study. In this study we compared the effects of kinesiotaping versus friction massage (a common method of trigger point treatment) on latent trigger points in the upper trapezius muscle.

## Methods

This was a parallel single-blind randomized clinical trial (Registration ID: IRCT2016080126674N3) in which the assessor was unaware of the participants’ group allocation. A total of 58 male students participated in this study. Participants were recruited at 8 different schools of Shiraz University of Medical Sciences with a stratified sampling method. The number of participants selected from each school was determined in proportion to the student population. This study was done in 2016.

Inclusion criteria for this study were the presence of a latent trigger point in the proximal third of the upper trapezius muscle, and age between 18 and 30 years. To match participants across groups, the trapezius muscle was examined for any trigger points and only participants with latent trigger point in the proximal third entered the study. Participants with any other latent trigger points, any active trigger points, musculoskeletal disorders in the neck or shoulder(s), cervical disk herniation, or any radicular pain in the upper extremity were excluded from the study. The participants signed an informed consent form before entering the study. They were randomly assigned to two different treatment groups: kinesiotaping or friction massage. We used a random assignment method in which each participant received a random number; participants with odd numbers were allocated to one group and participants with even numbers were allocated to the other group.

The outcome measures in this study consisted of the pressure pain threshold at the trigger point site and grip strength. Pressure pain threshold was recorded with a pressure algometer (Wagner, USA) and grip strength was recorded with a Collin dynamometer (COMED, France). These instruments are reliable and valid [9, 10].

In order to record pressure pain threshold, the algometer was placed vertically on the trigger point and pressed into the body surface, and participants were asked to report the moment of pain sensation. At this moment the threshold (kg/cm^2^) was recorded from the algometer screen. This procedure was repeated 3 times and the mean value was recorded as the participant’s pressure pain threshold. To record grip strength, participants were seated on a chair with their forearm on the table. The elbow was in 90 degrees of flexion and the shoulder was in 30 degrees of abduction. Participants were asked to hold the dynamometer in their hand and grip it with maximal power. Grip strength was recorded as the number (kg) from the dynamometer screen. This procedure was repeated 3 times and the mean value was recorded as grip strength. This initial evaluation was done by researcher 1 (S.H.).

**Fig. 1:**
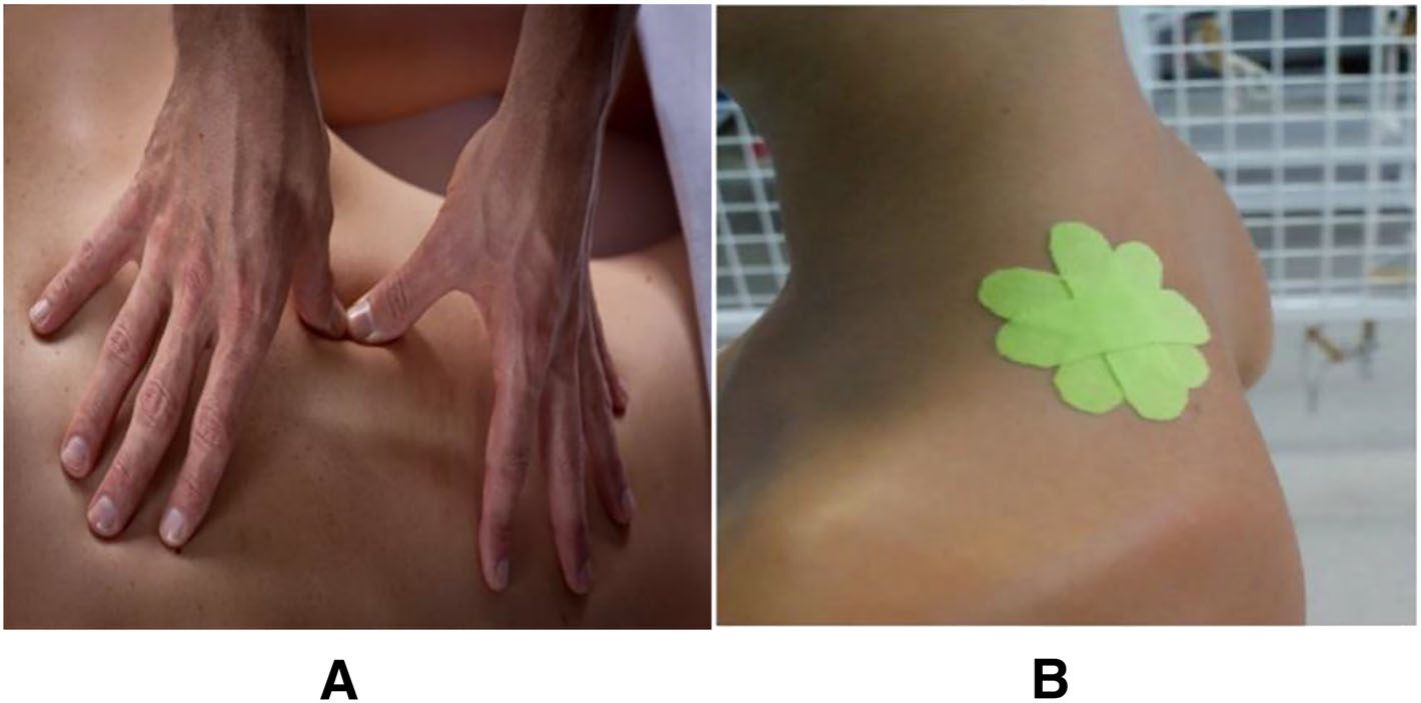
Friction massage (**a**) and kinesiotaping (**b**)

Treatments were given by researcher 2 (I.R.). Participants in group 1 received friction massage according to Trampas et al. [11] and those in group 2 received space correction kinesiotaping according to Kumbrink [12] at the upper trapezius trigger point. Friction massage was applied in 3 sessions on 3 successive days and kinesiotape was used for 72 h. One hour after the last session of friction massage or after removal of the kinesiotape, the pressure pain threshold and grip strength were measured again by researcher 1 (Fig. 1).

### Statistical analysis

The sample size was determined to be 29 cases in each group based on a previous study (α = 0.05, 1-β = 0.85) [2]. Descriptive and analytic statistics were used. Normality of the data was verified with the Kolmogorov–Smirnov test. Data analysis was conducted with SPSS v. 16. Independent and paired t-tests were used for analysis. The pretreatment pressure pain threshold differed significantly between the two groups, so we used ANCOVA for between-group comparisons. The significance level was set at 0.005.

## Results

**Fig. 2:**
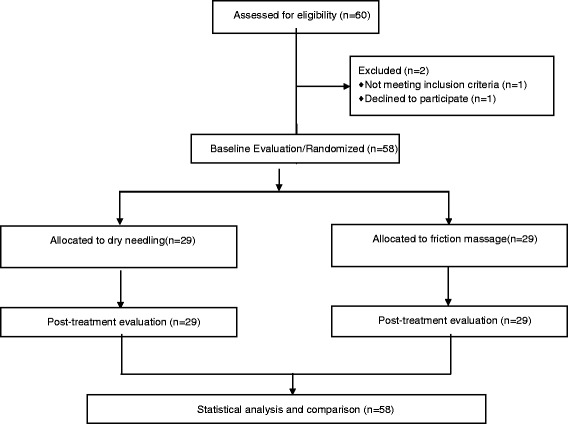
Flowchart of the enrollment and testing procedures

The eligibility criteria of the participants are summarized in Fig. 2. The demographic characteristic of participants are shown in Table 1. There were no significant differences between the two groups in mean age, height, weight, and pretreatment grip strength (*P* > 0.05), but pretreatment pressure pain threshold was significantly different between group (*P* < 0.05), so the ANCOVA test was used for between-group analysis of this variable (P of interaction × covariate = 0.26). The pressure pain threshold decreased significantly after both friction massage (2.66 ± 0.89 to 2.25 ± 0.76; *P* = 0.02; %95 of CI = 0.746, 0.07) and kinesiotaping (2.00 ± 0.74 to 1.71 ± 0.65; *P* = 0.01; %95 of CI = 0.497, 0.065). Grip strength increased significantly after friction massage (40.78 ± 9.55 to 42.17 ± 10.68; *P* = 0.03; %95 of CI = −0.143, −2.626); however, there was no significant posttreatment change in the kinesiotape group (39.72 ± 6.42 to 40.65 ± 7.3; *P* = 0.197; %95 of CI = 0.514, − 2.388). There were no significant differences between the two study groups in pressure pain threshold or grip strength after treatment (Table 2).

**Table 1:** Characteristics of participants at baseline

Variable Group	Site of involvement		Age (years)	Height (cm)	Weight (kg)	Pressure pain threshold	Grip strength
	Right	Left					
Friction massage	40.9%	59.1%	21.63 ± 1.59	176.95 ± 6.19	68.22 ± 9.29	2.66 ± 0.89	40.78 ± 9.55
Kinesiotape	40%	60%	22.04 ± 1.76	178.28 ± 6.10	71.28 ± 12.05	2.00 ± 0.74	39.72 ± 6.42

**Table 2:** Comparison of pressure pain threshold and grip strength between the two study groups

Variable	Treatment group (Mean ± SD)		*P*
	Friction massage	Kinesiotape	
Pressure pain threshold	2.10 ± 0.11**	1.87 ± 0.11**	0.53
Grip strength	42.17 ± 10.68**	40.65 ± 7.30	0.66

**Significant at *P* < 0.05 (within-group comparison with paired t-test)

## Discussion

The results of this study showed that the friction massage and kinesiotaping had identical short-term effects on latent trigger points in the upper trapezius. In both groups, pressure pain threshold decreased after treatment. In the friction massage group, grip strength increased significantly after treatment. The low effect size (0.03) for pressure pain threshold shows that there were no differences between these two treatment methods; however, the effect size covariate (0.28) indicated that the significant difference between study groups at baseline may have affected the results of this study.

The decrease in pressure pain threshold in both groups may indicate that these interventions not only failed to ameliorate latent trigger points, but may have stimulated these points or attracted participants’ attention to their pain. This result was contrary to our expectations, and contrasts with the results published by Chao et al., Halski et al. and Haran et al. [2, 13, 14].

Xu et al. claimed that short-term mechanical nociceptive stimulation of trigger points can cause pain centralization [15]. Zhang et al. stated that painful irritation of latent trigger points may increase sympathetic activity and reduce skin blood flow, which in turn may favor the accumulation of substances that facilitate the perception of pain in the area [16]. In the present study, both interventions may be considered painful stimuli to latent trigger points. Despite the small number of intervention sessions we used, they were sufficient to stimulate latent trigger points. As a result, our participants’ latent trigger points were stimulated, activated and apparently became active trigger points, and these phenomena decreased the pressure pain threshold. Another reason for lower pressure pain threshold after treatment may have been the increased attention to trigger points and pain in our study participants.

Grip strength in our participants increased only after friction massage. This parameter is affected by proximal stability and is dependent on shoulder and scapular stability. The upper trapezius, lower trapezius and serratus anterior are key muscles in scapular stability, and impaired balance among these muscles can disrupt stability [7, 8]. Treatment of the upper trapezius muscle can result in the release of hand and wrist extensor muscles through the release of the superficial back arm line. Given the key role of the wrist extensor muscles in wrist stability and hence better grip, intervention to treat trigger points in the upper trapezius can improve grip strength [17].

According to previous studies, taping may reduce or increase muscle strength through neuroinhibition or neurofacilitation [18–20]. Although taping is expected to cause tactile inputs to the central nervous system, these inputs may not be strong enough to change motor neuron excitability in the central nervous system. This putative mechanism is consistent with findings published by Halski et al. and Cools et al., who reported that taping was unable to change muscle electrical activity and tonicity [14, 21].

The improvement in grip strength after friction massage in the present study may be attributable to its deeper effects compared to taping. Friction massage is applied directly on muscle fibers, whereas taping affects tactile efferents. According to previous studies of interventions to enhance grip strength, medium to high pressure is required during shoulder and arm massage [22]. The pressure created by taping may thus be too weak to increase grip strength. It is noteworthy that mean grip strength did not differ significantly between our two study groups after the interventions.

One of the main advantages of this study is our assessment of grip strength. Previous research on the effect of treatment on latent trigger points in the upper trapezius has been published by Zhang et al. [16], Trampus et al. [11] and Sarrafzadeh et al. [23]; however, none of these studies considered upper limb function. In 2012, Lee et al. used the Constant–Murley Scale for functional assessment in patients with myofascial pain syndrome [24]. In the present study we assessed limb function as grip strength rather than as responses to a questionnaire.

The main limitation of this study is that our population was limited to male university students 18 to 30 years old. Also, we did not record other symptoms of latent trigger points such as fatigue and range of motion limitation. Other limitations of this study were the short duration of treatment and the absence of follow-up data. Ways to avoid these potential shortcomings should be considered in the design of future studies.

## Conclusions

Overall, the results of this study showed that friction massage and kinesiotaping had the same effects on latent trigger points in the upper trapezius. Three sessions of both interventions were unable to alleviate latent trigger points. More prolonged interventions over more sessions may yield different results, and this possibility should be considered in future research on the management of latent trigger points in the upper trapezius.

## Abbreviation

SUMS: Shiraz University of Medical Sciences
